# Associations of Word Recognition in Noise with Perceived Hearing Handicap and Quality of Life in Older Adults with Sensorineural Hearing Loss

**DOI:** 10.3390/audiolres16050143

**Published:** 2026-09-25

**Authors:** Ebru Sönmez, Çağlar Batman

**Affiliations:** 1 Department of Audiology, Institute of Graduate Studies, Istanbul University-Cerrahpaşa, Istanbul 34320, Türkiye; 2 Department of Speech and Language Therapy, Faculty of Health Sciences, Haliç University, Istanbul 34060, Türkiye; 3 Department of Otorhinolaryngology-Head and Neck Surgery, Faculty of Medicine, Marmara University, Istanbul 34854, Türkiye

**Keywords:** sensorineural hearing loss, noise, signal-to-noise ratio, word recognition, hearing handicap, quality of life, older adults

## Abstract

Background/Objectives: Pure-tone audiometry and word recognition scores (WRS) obtained in quiet may not fully reflect everyday hearing difficulties. This study examined how WRS across different listening conditions relate to perceived hearing handicap and quality of life in older adults with sensorineural hearing loss, and whether they explain perceived handicap beyond demographic and audiological variables. Methods: Seventy-nine older adults with sensorineural hearing loss were included. WRS were assessed in quiet and at +10 dB and 0 dB signal-to-noise ratios (SNRs) using ipsilateral speech noise. Perceived hearing handicap and quality of life were evaluated using the Turkish Hearing Handicap Inventory for the Elderly (HHIE) and World Health Organization Quality of Life-Older Adults Module (WHOQOL-OLD), respectively. Listening conditions were compared using Friedman and Bonferroni-corrected Wilcoxon tests. Associations were examined with Spearman correlations, and hierarchical regression assessed the independent contribution of WRS after adjustment for age, pure-tone average, hearing loss duration, and tinnitus. Results: WRS decreased from 88% in quiet to 72% at +10 dB SNR and 32% at 0 dB SNR (*p* < 0.001). Lower WRS was associated with greater hearing handicap, most strongly at 0 dB SNR (r = −0.491, *p* < 0.001), while no significant association was observed with overall quality of life. Both speech-in-noise conditions explained a similar amount of additional variance in HHIE total scores beyond demographic and audiological variables (+10 dB SNR, ΔR^2^ = 0.037, *p* = 0.054; 0 dB SNR, ΔR^2^ = 0.042, *p* = 0.039). Conclusions: Word recognition in noise reflected perceived hearing handicap better than performance in quiet. Speech recognition in noise may complement hearing-specific self-report measures in evaluating everyday hearing difficulties.

## 1. Introduction

Hearing loss is a common sensory impairment that can occur at any age. In older adults, its most common form is age-related hearing loss (presbycusis), a progressive and usually bilateral, symmetrical sensorineural loss [1]. Global Burden of Disease data show that its prevalence is rising among people aged 60 and over and will continue to increase as populations age [2]. However, its impact extends well beyond a change in hearing thresholds, affecting communication, social participation and quality of life. When it is left untreated, it makes verbal communication harder, may contribute to social isolation and depression, and is regarded as a modifiable risk factor for cognitive decline and dementia [3]. For this reason, its effects are increasingly judged not by hearing thresholds alone, but by how well the person functions in everyday listening, where the most demanding situation is understanding speech against background noise.

This age-related loss predominantly affects the high frequencies. Although high-frequency consonants contribute relatively little to the overall energy of speech, they provide important acoustic cues for distinguishing phonemes and identifying words. Reduced audibility of these cues may therefore impair speech recognition, particularly for monosyllabic words [4,5]. As a result, the most common complaint is not that patients cannot hear speech, but that they cannot follow what is said, especially against noise or reverberation [6,7]. In routine practice, however, the word recognition score (WRS) is measured in quiet, and quiet performance does not reliably predict performance in noise. Poor scores in quiet usually go together with poor scores in noise, but good scores in quiet do not guarantee good scores in noise [8]. This is because speech-in-noise ability also depends on cognitive and non-auditory factors, not on pure-tone thresholds alone [9]. Testing speech in noise, and at several signal-to-noise ratios (SNRs), grades task difficulty and shows these individual differences more clearly [10,11]. For this reason, speech-in-noise testing is increasingly recommended in routine audiological batteries, because it is closer to real-life listening [12]. On their own, pure-tone audiometry and a quiet WRS may be limited for interpreting the everyday impact of hearing loss [13].

Yet even a speech test in noise is still a clinician-measured index. It does not show how difficult the person feels their hearing problem to be. This perceived impact is multidimensional. It covers daily communication, withdrawal from social interaction, emotional distress and reduced quality of life. Self-report measures can therefore add to objective testing at two levels. Hearing-specific scales capture perceived hearing handicap, while general quality-of-life scales reflect broader effects on well-being. These two perspectives have increasingly been examined together. Recent large-sample clinical studies show that when speech is presented in noise, performance relates to how much difficulty patients report, whereas word recognition measured in quiet shows little or no such relationship [14,15]. On this basis, speech-in-noise testing has been proposed for inclusion in the routine audiological battery [8,12].

The relationship between speech-in-noise performance and perceived hearing handicap is itself not new. Word discrimination in quiet has long been shown to correlate only weakly with self-reported handicap [16], whereas speech recognition in noise relates to HHIE scores even after audibility is controlled [17], and large epidemiological samples have linked word recognition in quiet and in competing speech to HHIE-S scores [18]. However, most of this evidence comes from adaptive, sentence- or digit-based tests that are scored as a signal-to-noise ratio loss and are paired with self-report scales of perceived ability such as the SSQ. What remains far less clear is whether the routine monosyllabic word recognition test used in everyday clinical practice, when it is simply extended into fixed, graded SNR noise rather than replaced by an adaptive sentence- or digit-based test, carries the same information, and whether it adds to the HHIE beyond routine demographic and audiological variables. It is also unclear whether this relationship is specific to hearing-focused self-report or extends to general quality of life in older adults with sensorineural hearing loss.

This study therefore examined the association of WRS, measured in quiet and at different SNRs, with perceived hearing handicap and quality of life in this population, and assessed whether WRS helps to explain perceived handicap beyond demographic and audiological variables.

## 2. Materials and Methods

This cross-sectional study was carried out at the Department of Audiology and Speech Disorders, Division of Otorhinolaryngology, Faculty of Medicine, Marmara University. The study protocol was approved by the Ethics Committee of the Faculty of Medicine, Marmara University (protocol no. 09.2019.038), and the study was conducted in accordance with the principles of the Declaration of Helsinki. All participants were informed about the aim and scope of the study, and written informed consent was obtained from each of them.

### 2.1. Participants

Eligibility was assessed using acoustic immittance measurements, pure-tone audiometry and the Mini-Mental State Examination (MMSE) [19]. Participants were included if they met all of the following criteria: aged 60 years or over; no conductive pathology in the outer or middle ear; a Type A tympanogram on tympanometry with a 226 Hz probe tone; sensorineural hearing loss; a four-frequency air-conduction average (500, 1000, 2000 and 4000 Hz) in the better-hearing ear between 16 and 70 dB HL, corresponding to a mild to moderately severe loss on the Clark classification [20]; a history of hearing loss of at least one year; no current hearing aid use; an MMSE score of 24 or above; no neurological or speech disorders; and Turkish as their native language.

### 2.2. Procedure

Before testing, demographic information (age, gender, educational level, marital status and employment status) and audiological information (duration of hearing loss, presence of tinnitus and history of hearing aid use) were collected through a face-to-face interview. The duration of hearing loss was recorded in years, as the time from when the participant first noticed the difficulty to the date of assessment. The presence of tinnitus was recorded as present or absent, based on self-report. Cognitive function was assessed with the MMSE. The MMSE was used solely as a screening tool to exclude possible clinically relevant cognitive impairment and was not intended as a quantitative measure of individual cognitive performance. Participants then completed the Turkish Hearing Handicap Inventory for the Elderly (HHIE), to assess perceived hearing handicap, and the Turkish version of the World Health Organization Quality of Life Instrument, Older Adults Module (WHOQOL-OLD), to assess quality of life. Acoustic immittance testing, pure-tone audiometry and speech audiometry were then performed, in that order.

#### 2.2.1. Audiological Assessment

Immittance measurements were performed with an Interacoustics AT235 device (Interacoustics A/S, Middelfart, Denmark). Middle ear function was assessed by tympanometry with a 226 Hz probe tone, and a Type A tympanogram was taken to indicate normal middle ear function. Ipsilateral and contralateral acoustic reflex thresholds were also recorded at 500, 1000 and 2000 Hz. Pure-tone audiometry was carried out in a sound-insulated test room with an Interacoustics Equinox AC-440 clinical audiometer (Interacoustics A/S, Middelfart, Denmark). Air-conduction thresholds were measured with TDH 39 (Telephonics Corporation, Farmingdale, NY, USA) from 250 to 8000 Hz, and bone-conduction thresholds with a B-71 bone vibrator (RadioEar, Middelfart, Denmark) from 500 to 4000 Hz. Sensorineural hearing loss was confirmed when air- and bone-conduction thresholds were consistent and the air-bone gap did not exceed 10 dB at 500, 1000, 2000 and 4000 Hz. The pure-tone average (PTA) was calculated as the arithmetic mean of the air-conduction thresholds at these four frequencies for each ear. PTA values were reported separately for the right and left ears, as well as for the better- and worse-hearing ears. The degree of hearing loss was also classified for each ear. Hearing loss was considered symmetric when both ears fell within the same hearing-loss severity category and asymmetric when the ears were classified into different severity categories.

All speech tests were administered by the same researcher using live speech, with the peak levels of the words kept at 0 VU on the VU meter. The speech reception threshold (SRT) was determined with Turkish three-syllable word lists routinely used in clinical practice. The WRS was assessed with Turkish phonemically balanced word lists of 25 monosyllabic words [21]. A different list was used for each listening condition to reduce the effects of learning and familiarity, and the order of the conditions was randomized across participants to reduce order and fatigue effects. Speech was presented at the most comfortable level (MCL): the starting level was set 30 to 40 dB above the SRT (30 to 40 dB SL), and it was then confirmed verbally that this was the most comfortable level for the participant. Each word repeated correctly was scored as 4 percent, so the WRS was the percentage of correct responses, reaching 100 percent for a participant who repeated all 25 words correctly.

The WRS was measured in quiet and under +10 dB and 0 dB SNR conditions. For the noise conditions, the internal speech noise of the Interacoustics Equinox AC-440 audiometer was used, and the speech and noise were presented ipsilaterally to the same ear. The speech level was kept constant while the noise level was adjusted to set the SNR: the noise was 10 dB below the speech level for the +10 dB SNR condition, and equal to the speech level for the 0 dB SNR condition. The better-hearing ear was defined as the ear with the lower PTA across 500, 1000, 2000 and 4000 Hz, and the WRS values used in the analyses were those from this ear in the quiet, +10 dB SNR and 0 dB SNR conditions. These SNRs were chosen to represent progressively more difficult listening conditions within the same monosyllabic word recognition paradigm [22]. For all WRS analyses, the better-hearing ear was defined according to the four-frequency PTA. When PTA values were identical between ears, the ear subjectively perceived by the participant as having better hearing was selected.

#### 2.2.2. Self-Reported Scales

Perceived hearing handicap was assessed with the HHIE, and quality of life with the Turkish version of the WHOQOL-OLD [23,24]. The HHIE is a 25-item self-report scale that measures the emotional and social/situational effects of hearing loss on the daily lives of older adults through an emotional subscale (13 items) and a social/situational subscale (12 items). Each item is answered as “yes” (4 points), “sometimes” (2 points) or “no” (0 points), giving a total score from 0 to 100, where higher scores indicate greater perceived hearing handicap. A total of 0 to 16 indicates no handicap, 18 to 42 a mild to moderate handicap, and 44 or above a significant handicap [25]. In the Turkish validity and reliability study, internal consistency was high (Cronbach’s α = 0.95) [23].

The WHOQOL-OLD is a complementary module of the WHOQOL-BREF. It is a 24-item self-report scale that assesses quality-of-life features specific to older adults across six subdimensions: sensory abilities, autonomy, past, present and future activities, social participation, death and dying, and intimacy. Each subdimension has four items rated on a 5-point Likert-type scale [24]. Seven negatively worded items (items 1, 2, 6, 7, 8, 9 and 10), which belong to the sensory abilities and death and dying subdimensions, were reverse-coded before analysis so that higher scores always indicated better quality of life. Each subdimension score was the sum of its four items (range 4 to 20), and the total score was the sum of the six subdimensions (range 24 to 120), with higher scores indicating better quality of life. The validity and reliability of the Turkish version have been shown in Turkish adults aged 65 and over [24].

### 2.3. Statistical Analysis

The distribution of the variables was checked visually and analytically. Because these variables were not normally distributed, they were summarized as median, interquartile range (IQR) and minimum–maximum, while categorical variables were summarized as frequencies and percentages. The WRS values in the three listening conditions (quiet, +10 dB SNR and 0 dB SNR) were compared with the Friedman test, with Kendall’s W as the effect size. When the overall test was significant, pairwise comparisons were made with Wilcoxon signed-rank tests and Bonferroni correction. Spearman correlations were used to examine the associations of WRS in each listening condition with HHIE total, HHIE emotional, HHIE situational, WHOQOL-OLD total and WHOQOL-OLD subscale scores. Hierarchical multiple linear regression was used to test whether WRS in the different listening conditions explained additional variance in the HHIE total score beyond demographic and audiological factors. The HHIE total score was the dependent variable. The base model included age, PTA, duration of hearing loss and tinnitus status. In the second step, each WRS variable (quiet, +10 dB SNR and 0 dB SNR) was added separately. Model improvement was assessed with the change in R^2^, the F-change statistic and its *p* value. Regression coefficients were reported as unstandardized coefficients with standard errors, t values and *p* values. Multicollinearity was assessed with variance inflation factors (VIF), and the regression assumptions were checked by visual inspection of residual and normal Q-Q plots. To account for multiple testing across the three correlated WRS models, a permutation-based max-F procedure was performed as a sensitivity analysis. As a sensitivity analysis for ceiling effects, the correlation and regression analyses were repeated after excluding participants scoring at or above 96% in word recognition in quiet). A *p* value below 0.05 was considered statistically significant. All analyses were performed in R (version 4.2.2) [26].

### 2.4. Sample Size

An a priori sample-size calculation was performed using G*Power (version 3.1.9.6) for the hierarchical regression analysis. The analysis was specified as an F test for linear multiple regression, fixed model, R^2^ increase, with a medium effect size (f^2^ = 0.15), an α level of 0.05, and statistical power of 80%. One predictor was specified as the tested predictor (WRS), with five predictors in the full model, including age, pure-tone average, duration of hearing loss, tinnitus status, and WRS. The minimum required sample size was 55 participants. To allow for a more robust analysis, 79 participants were ultimately included in the study.

## 3. Results

### 3.1. Participant Characteristics

A total of 79 participants were included in the study. The median age was 66 years (IQR: 62–70; range: 60–78). Of the participants, 32 were female (40.5%), and 47 were male (59.5%). Most participants were married (88.6%) and retired or unemployed (88.6%). Tinnitus was present in 55 participants (69.6%) and absent in 24 participants (30.4%).

The median duration of hearing loss was 3 years (IQR: 1–7; range: 1–30). The median pure-tone average was 27.5 dB HL (IQR: 18.75–38.12; range: 16.25–68.75). Median WRS decreased across listening conditions, from 88% in quiet to 72% at +10 dB SNR and 32% at 0 dB SNR. The median HHIE total score was 28 (IQR: 12–50; range: 0–92). Detailed demographic, clinical, audiological, hearing handicap, and quality-of-life characteristics are presented in Table 1.

### 3.2. Comparison of WRS Across Listening Conditions

The Friedman test showed a significant difference in WRS across the three listening conditions, Friedman χ^2^(2) = 157.50, *p* < 0.001, with a very large effect size, Kendall’s W = 0.997. Median WRS decreased progressively as listening difficulty increased, from quiet conditions [88% (IQR: 80–96)] to +10 dB SNR [72% (IQR: 54–84)] and 0 dB SNR [32% (IQR: 16–50)]. Bonferroni-corrected post hoc comparisons confirmed that all pairwise differences were statistically significant: quiet versus +10 dB SNR, quiet versus 0 dB SNR, and +10 dB SNR versus 0 dB SNR, all adjusted *p* < 0.001 (see Figure 1 and Supplementary Materials Table S1).

### 3.3. Correlations Between WRS, Hearing Handicap, and Quality of Life

Spearman correlation analyses showed significant negative correlations between WRS values and HHIE total, emotional, and situational scores. For the HHIE total score, the correlation was weakest for WRS in quiet, r = −0.283, *p* < 0.05, and stronger for speech-in-noise conditions, particularly WRS at +10 dB SNR, r = −0.452, *p* < 0.001, and WRS at 0 dB SNR, r = −0.491, *p* < 0.001. A similar pattern was observed for the HHIE emotional subscale, with correlations ranging from r = −0.236 to r = −0.470, and for the HHIE situational subscale, with correlations ranging from r = −0.310 to r = −0.473.

In contrast, WRS values were not significantly correlated with the WHOQOL-OLD total score or most WHOQOL-OLD subscale scores. The only significant associations were observed for the WHOQOL-OLD sensory abilities subscale, which was positively correlated with WRS at +10 dB SNR, r = 0.283, *p* < 0.05, and WRS at 0 dB SNR, r = 0.313, *p* < 0.01. These findings indicate that poorer speech recognition, particularly under noise conditions, was associated with greater perceived hearing handicap. In contrast, its relationship with overall quality of life was limited (see Figure 2 and Figure 3).

A weak negative correlation was also observed between HHIE total and WHOQOL-OLD total scores (r = −0.229, *p* = 0.043), indicating that greater perceived hearing handicap was associated with slightly poorer overall quality of life. The distribution of this relationship is shown in Figure 4.

### 3.4. Hierarchical Regression Analyses Predicting HHIE Total Score

Hierarchical multiple linear regression analyses were conducted to determine whether WRS under different listening conditions explained additional variance in the HHIE total score beyond age, PTA, duration of hearing loss, and tinnitus status. The base model accounted for 25.8% of the variance in the HHIE total score, R^2^ = 0.258, adjusted R^2^ = 0.218.

Adding WRS in quiet did not improve model fit, ΔR^2^ = 0.001, F-change = 0.121, *p* = 0.729, indicating that word recognition performance under quiet conditions did not provide additional explanatory value beyond the base model. The two speech-in-noise conditions produced very similar increments in explained variance (WRS at +10 dB SNR, ΔR^2^ = 0.037, F-change = 3.836, *p* = 0.054; WRS at 0 dB SNR, ΔR^2^ = 0.042, F-change = 4.399, *p* = 0.039). Given that these values are almost identical and fall on either side of the conventional 0.05 threshold, they are interpreted together rather than as one significant and one non-significant effect. In the permutation-based max-F sensitivity analysis accounting for multiple testing across the three correlated WRS models, the additional contribution of WRS at 0 dB SNR remained statistically significant (adjusted *p* = 0.045), whereas WRS in quiet (adjusted *p* = 0.964) and WRS at +10 dB SNR (adjusted *p* = 0.116) remained non-significant.

Across the final models, duration of hearing loss remained a significant predictor of HHIE total score. PTA was significant only in the model including WRS in quiet, B = 0.608, SE = 0.296, *p* = 0.043, whereas its effect was attenuated when speech-in-noise variables were included. Age and tinnitus status were not independently associated with HHIE total score in any model. Variance inflation factor values ranged from 1.019 to 2.109, indicating no evidence of problematic multicollinearity (see Table 2). Overall, these findings suggest that speech recognition performance under challenging listening conditions, particularly at 0 dB SNR, is more closely related to perceived hearing handicap than word recognition performance measured in quiet. Relative to the unexplained variance, this increment corresponds to a small effect (Cohen’s f^2^ ≈ 0.06). In the ceiling sensitivity analysis it corresponded to a medium effect (f^2^ ≈ 0.21).

Word recognition in quiet showed a ceiling effect (39.2% scored ≥ 96%), which was rare at +10 dB SNR and absent at 0 dB SNR. In a sensitivity analysis excluding these participants (n = 48), the correlations strengthened, most significantly for quiet (HHIE total, r = −0.283 to −0.478), and both speech-in-noise conditions predicted HHIE total (both *p* = 0.005), whereas quiet did not (Supplementary Materials Tables S2 and S3).

## 4. Discussion

This study examined how word recognition, assessed in quiet and in noise, relates to perceived hearing handicap and quality of life in older adults with sensorineural hearing loss. The analyses yielded two main findings. First, word recognition declined significantly as the listening task became more demanding, decreasing from quiet to +10 dB SNR and again to 0 dB SNR. Second, it was performance in noise, rather than in quiet, that corresponded to the degree of hearing handicap reported by participants. In the hierarchical models, the score at 0 dB SNR accounted for additional variance in the HHIE total score beyond age, pure-tone average, duration of hearing loss and tinnitus. In addition, the score at 10 dB SNR also provided borderline significance. The most demanding listening condition therefore provided information about perceived handicap that pure-tone audiometry alone did not capture.

This pattern is consistent with a broader shift in how perceived hearing handicap is conceptualized. The base model, derived solely from age, pure-tone average, duration of hearing loss and tinnitus, explained approximately one quarter of the variance in the HHIE total (25.8%), leaving most interindividual differences unexplained. Pure-tone average was a significant predictor only while word recognition in quiet remained in the model, and its contribution diminished once the speech-in-noise scores were introduced. Thus, thresholds index the degree of hearing loss but do not fully represent the difficulty an individual encounters in everyday communication. A growing body of evidence converges on the same conclusion, which indicates that self-reported handicap is shaped not only by audiometric loss but also by factors such as speech understanding, frailty and mental health [27,28,29], and that the agreement between measured loss and reported difficulty is imperfect and varies with demographic and clinical characteristics [30,31]. Viewed against this background, the limited explanatory reach of the base model is to be expected rather than surprising.

The correlation analyses yielded a consistent pattern. The association between word recognition and perceived handicap was weakest in quiet (r = −0.283) and strengthened progressively as noise was introduced, reaching r = −0.452 at +10 dB SNR and r = −0.491 at 0 dB SNR. This gradient is consistent with, and can be situated between, two long-standing observations. Early work reported that word discrimination measured in quite correlated only weakly with self-reported handicap, more weakly even than pure-tone sensitivity [16], whereas more recent evidence has shown that speech recognition in noise relates to HHIE scores even after differences in audibility are taken into account [17]. Large epidemiological data have likewise linked word recognition, assessed both in quiet and in competing speech, to screening HHIE scores in older adults [18]. Our findings extend this literature by showing that the same gradient emerges within a single, clinically routine paradigm: as the identical monosyllabic test is moved from quiet into fixed +10 and 0 dB SNR noise, its correspondence with perceived handicap increases in step. As the test condition approaches the demands of everyday listening, it appears to correspond more closely to the handicap that individuals perceive. These observations accord with clinical evidence indicating that speech-in-noise performance relates to perceived difficulty more closely than word recognition in quiet, and that a quiet-only battery may fail to detect the difficulties some patients experience in noise [8,14]. A plausible explanation is that listening in noise engages more than hearing sensitivity, which reflects the combined influence of hearing loss, age-related changes in auditory processing and cognitive function [32,33]. The regression results followed the same gradient. The two speech in noise conditions yielded very similar increments (+10 dB SNR, ΔR^2^ = 0.037, *p* = 0.054; 0 dB SNR, ΔR^2^ = 0.042, *p* = 0.039). Rather than treating the former as null and the latter as robust on the basis of a narrow difference around the 0.05 threshold, we interpret both as pointing in the same direction, namely a small added contribution of speech-in-noise beyond audiometry. Consistent with this, both conditions contributed clearly and to a nearly identical degree once ceiling scores in quiet were removed (both ΔR^2^ = 0.12, *p* = 0.005).

Only the 0 dB SNR score reached significance, which explains a further 4.2% of the variance, with lower scores associated with greater handicap (B = −0.390, *p* = 0.039). Although statistically significant, this additional contribution is modest, and the 0 dB SNR score is therefore best regarded as a complementary measure rather than a standalone predictor of handicap. Because the three WRS conditions were each entered into separate regression models, sensitivity analyses were performed to evaluate the potential impact of multiple comparisons. The findings remained largely consistent; however, given that the incremental contribution of the 0 dB SNR condition was close to the conventional significance threshold, this result should be interpreted with caution. At 0 dB SNR, speech and noise are presented at equal levels, which may reveal the extent to which an individual can still exploit speech information under adverse conditions. In the present study, however, speech and a constant speech noise were delivered to the same ear, so this condition represents a controlled and simplified analogue of everyday listening rather than a model of a restaurant, a group conversation or sound arriving from multiple directions. Two further coefficients reinforce this cautious interpretation. Duration of hearing loss remained a significant predictor across all models, which possibly reflects the cumulative social and emotional burden of long-standing difficulty, although the cross-sectional design precludes any inference about the direction of this association. Age and tinnitus, by contrast, showed no independent association once the remaining variables were controlled, which does not imply that tinnitus is without effect, but rather that an independent contribution could not be demonstrated here. Collectively, these findings do not support abandoning the quiet word recognition score, but rather supplementing it with noise conditions and a hearing-specific self-report scale in the assessment and rehabilitation of older adults.

The incremental variance explained by speech-in-noise was modest, and speech-in-noise testing should therefore not be viewed as a strong predictor of perceived handicap on its own. Roughly 70% of the variance in HHIE scores was not accounted for by any of the audiological or demographic variables, which suggests that perceived hearing handicap is determined largely by non-audiological factors and that audiological measures, whether in quiet or in noise, can capture only part of it.

The findings for general quality of life were more limited. Word recognition was not significantly associated with the WHOQOL-OLD total score, and among its subscales only sensory abilities was related to performance at +10 dB SNR (r = 0.283) and 0 dB SNR (r = 0.313). These associations were weak and domain-specific and should therefore not be interpreted as evidence of a broader relationship between speech-in-noise performance and overall quality of life. The contrast with the HHIE likely reflects the different scope of the two instruments. The HHIE directly assesses the social and emotional consequences of hearing difficulty, whereas the WHOQOL-OLD covers broader domains such as autonomy, social participation, and intimacy, which are influenced by many factors beyond hearing. Accordingly, a hearing-specific instrument may be more sensitive to differences in auditory performance, whereas such effects may be attenuated in a broader quality-of-life measure. A comparable domain specificity has been reported in hearing aid users, in whom speech-in-noise performance related to circumscribed WHOQOL-BREF domains, such as social relationships, rather than to the global score [34], and in whom generic WHOQOL-BREF and HHIE scores diverged in their sensitivity to hearing aid benefit and to the severity of loss [35]. Our results extend these observations to untreated older adults assessed with the age-specific WHOQOL-OLD module, in whom word recognition in noise likewise tracked only the sensory abilities domain rather than overall quality of life. This interpretation is consistent with previous evidence showing that the impact of hearing loss on quality of life depends on the instrument and domain assessed [36,37]. Therefore, combining a general quality-of-life scale with a hearing-specific self-report instrument and a functional speech-in-noise test is likely to yield a more comprehensive clinical picture than any of these measures in isolation.

This study has several limitations. Its cross-sectional design precludes causal interpretation, and the sample was modest (n = 79) and selected: older adults with sensorineural hearing loss, no hearing aid use, and an MMSE of at least 24. The findings may therefore not generalize to people with normal hearing, hearing aid users, those with cognitive impairment, or other types of hearing loss. The models did not include depressive symptoms, frailty, comorbidity, social support, loneliness, education, or a detailed cognitive assessment (the MMSE served only as a screen), and they explained only a limited part of the HHIE variance. Because the incremental contribution of speech-in-noise was small in the full sample, its clinical predictive value is limited, and speech-in-noise testing is best used alongside self-report and broader biopsychosocial assessment rather than as a standalone predictor of handicap; incorporating factors such as auditory skills, frailty, and mental health in future work may help explain the large unexplained variance [29]. WRS was also based on the better-hearing ear, so the contribution of the poorer ear, interaural asymmetry, and binaural function may not be fully captured. Finally, speech was assessed only in quiet and at +10 and 0 dB SNR, with monosyllabic words and a constant ipsilateral speech noise; everyday listening is more complex, and future studies using adaptive tests, sentence-level materials, different noise types, spatial conditions, and recorded rather than live-voice stimuli would give a more complete and better-standardized account.

## 5. Conclusions

In older adults with sensorineural hearing loss, word recognition deteriorated substantially as background noise increased, and performance under noise was more closely associated with perceived hearing handicap than performance in quiet. Both speech-in-noise conditions contributed to a similar and modest degree to the HHIE total beyond demographic and audiological variables. Speech recognition in noise is therefore best regarded as a complement to, rather than a replacement for, established measures. Associations with general quality of life were limited and confined to the sensory abilities domain of the WHOQOL-OLD, which is consistent with the narrower, hearing-specific focus of the HHIE. Overall, these findings suggest that pure-tone audiometry and quiet word recognition may be usefully supplemented, in the clinical assessment of older adults, by speech-in-noise testing and a hearing-specific self-report instrument, so that everyday communication difficulty is more fully represented. Given the cross-sectional design and modest sample, these conclusions require confirmation in larger, longitudinal studies.

## Figures and Tables

**Figure 1:**
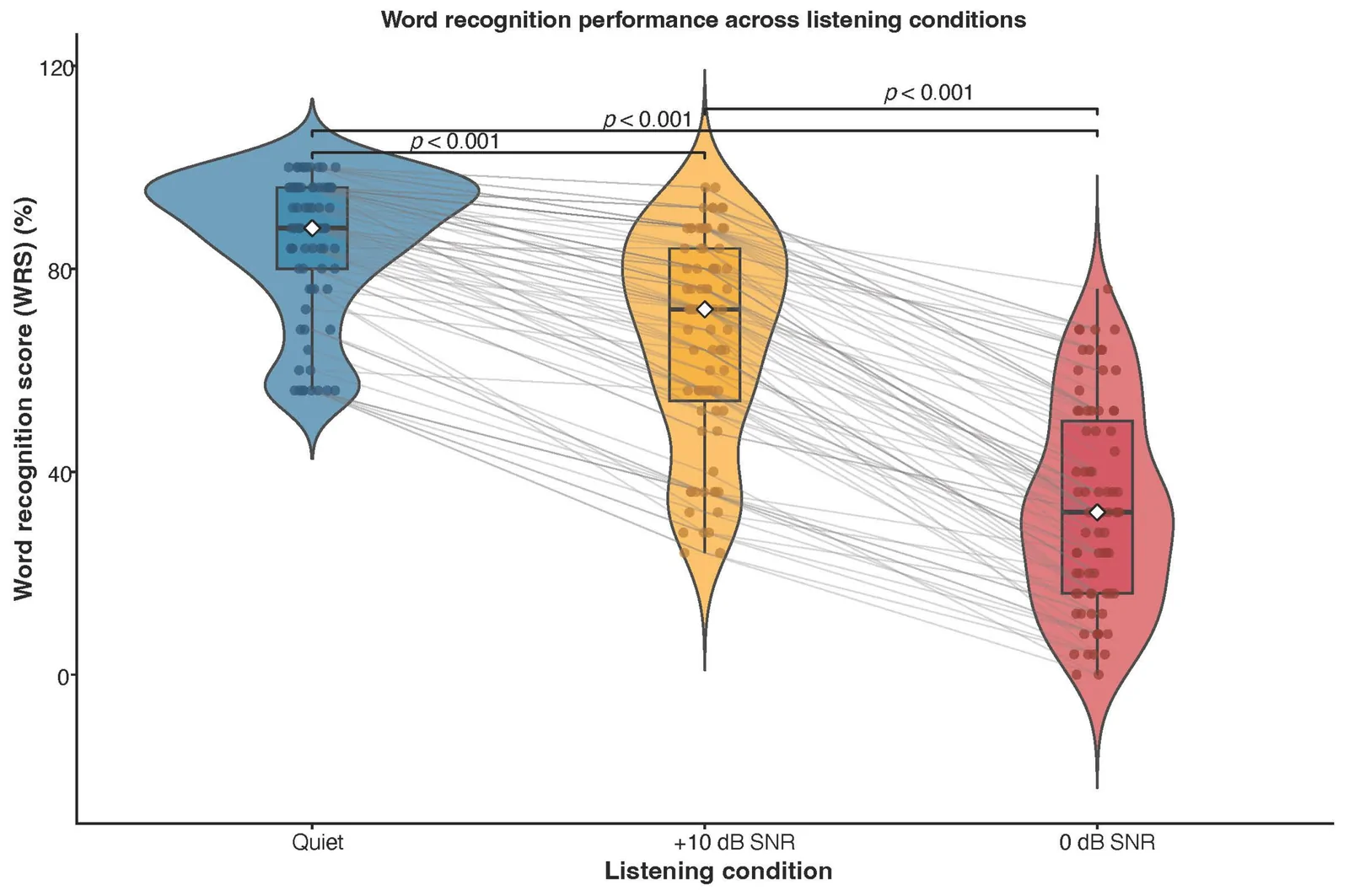
Word recognition performance across listening conditions. Violin plots showing the distribution of word recognition scores (WRS, %) in quiet, at +10 dB SNR, and at 0 dB SNR. Box plots indicate the median and interquartile range, white diamonds represent mean values, and individual data points are shown within each condition. Gray connecting lines represent within-subject changes across listening conditions.

**Figure 2:**
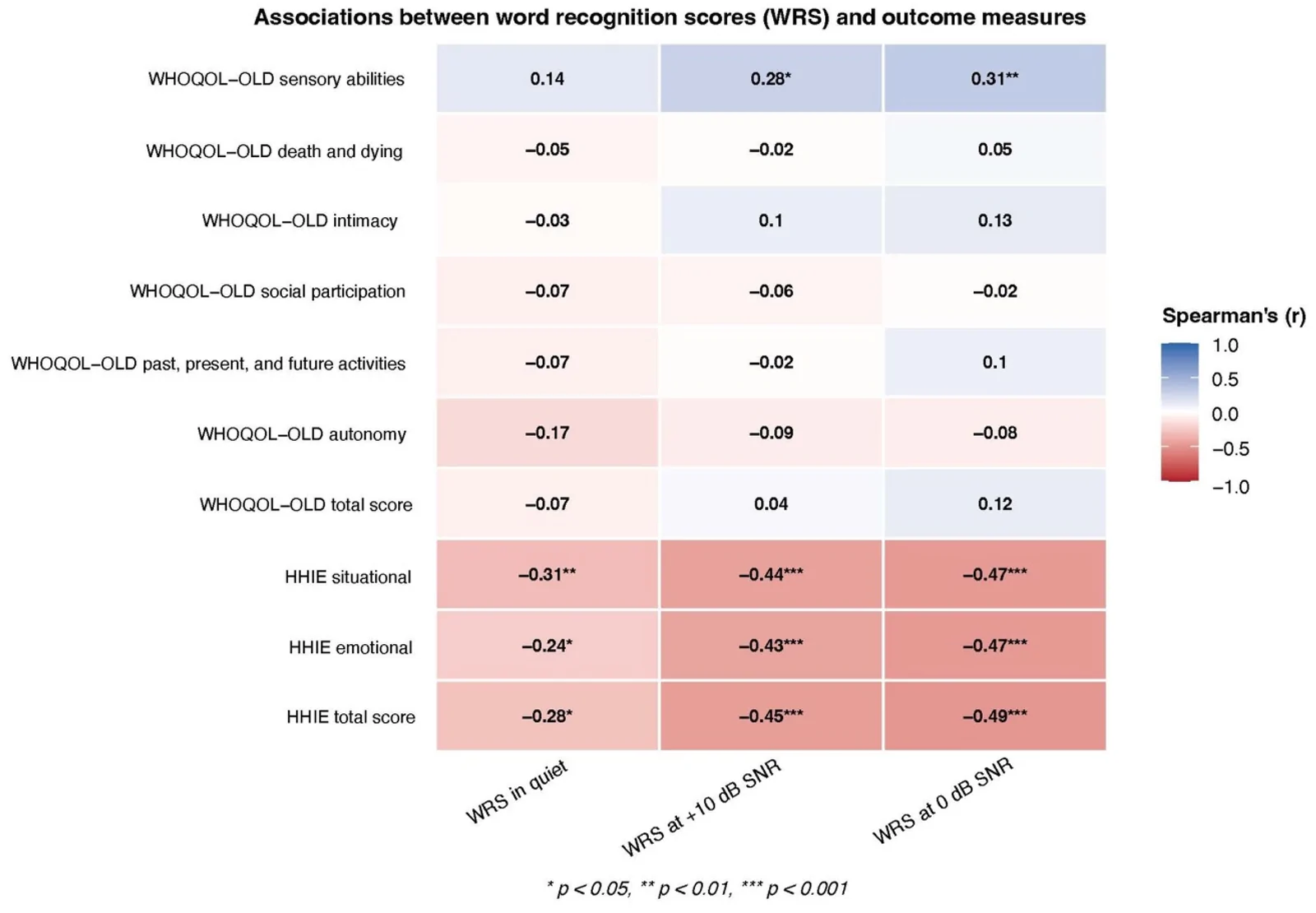
Associations between word recognition scores and outcome measures. Heatmap showing Spearman correlation coefficients between word recognition scores (WRS) across three listening conditions and quality-of-life/hearing handicap outcome measures. Blue tones indicate positive correlations, whereas red tones indicate negative correlations. Statistical significance is indicated as * *p* < 0.05, ** *p* < 0.01, and *** *p* < 0.001.

**Figure 3:**
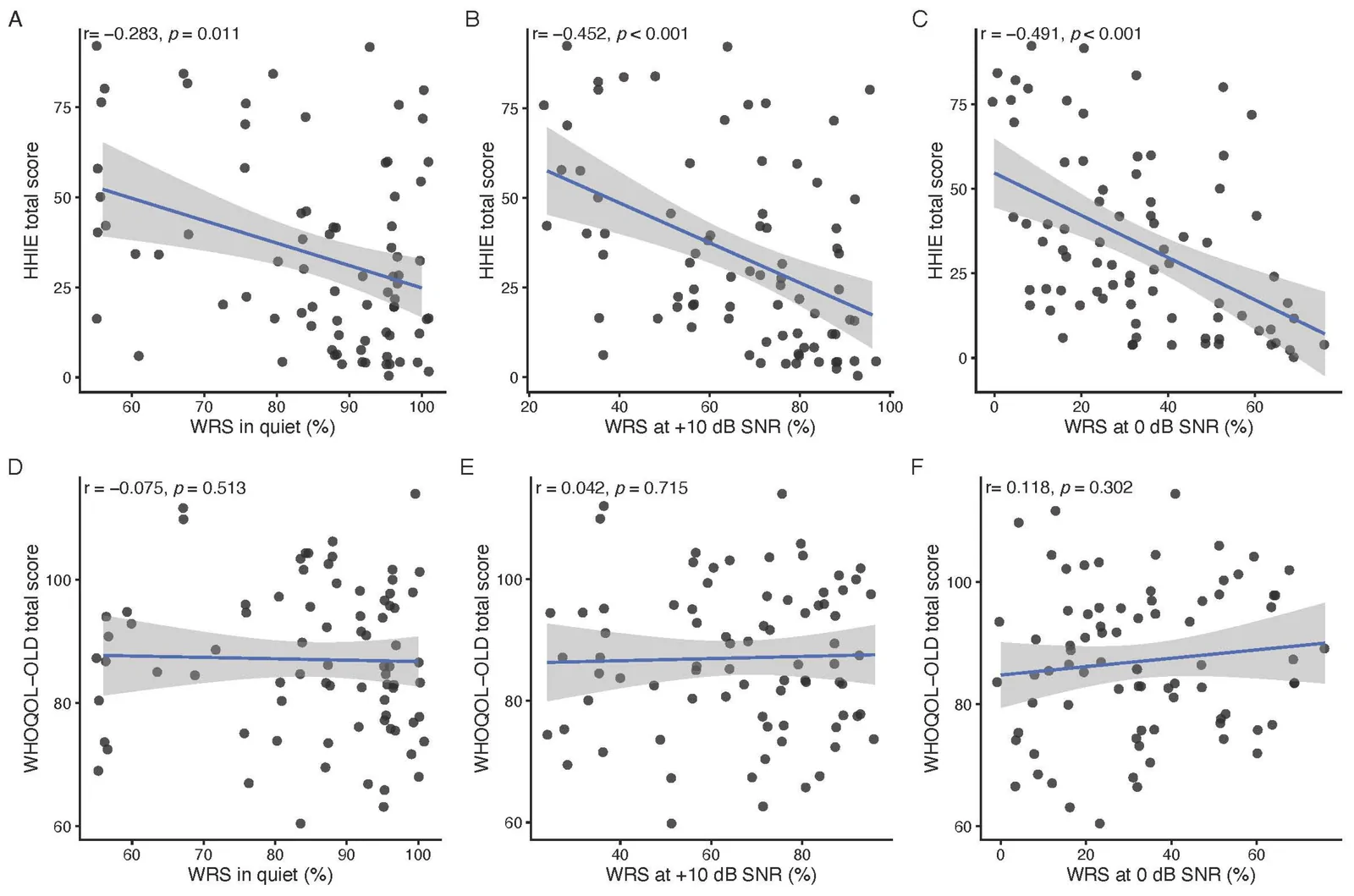
Scatterplots showing the associations of WRS in quiet, at +10 dB SNR, and at 0 dB SNR with HHIE total scores (**A**–**C**) and WHOQOL-OLD total scores (**D**–**F**). Spearman correlation coefficients (r) and corresponding *p*-values are shown. Lines represent fitted trends with 95% confidence intervals. The grey shaded area represents the 95% confidence interval around the fitted regression line.

**Figure 4:**
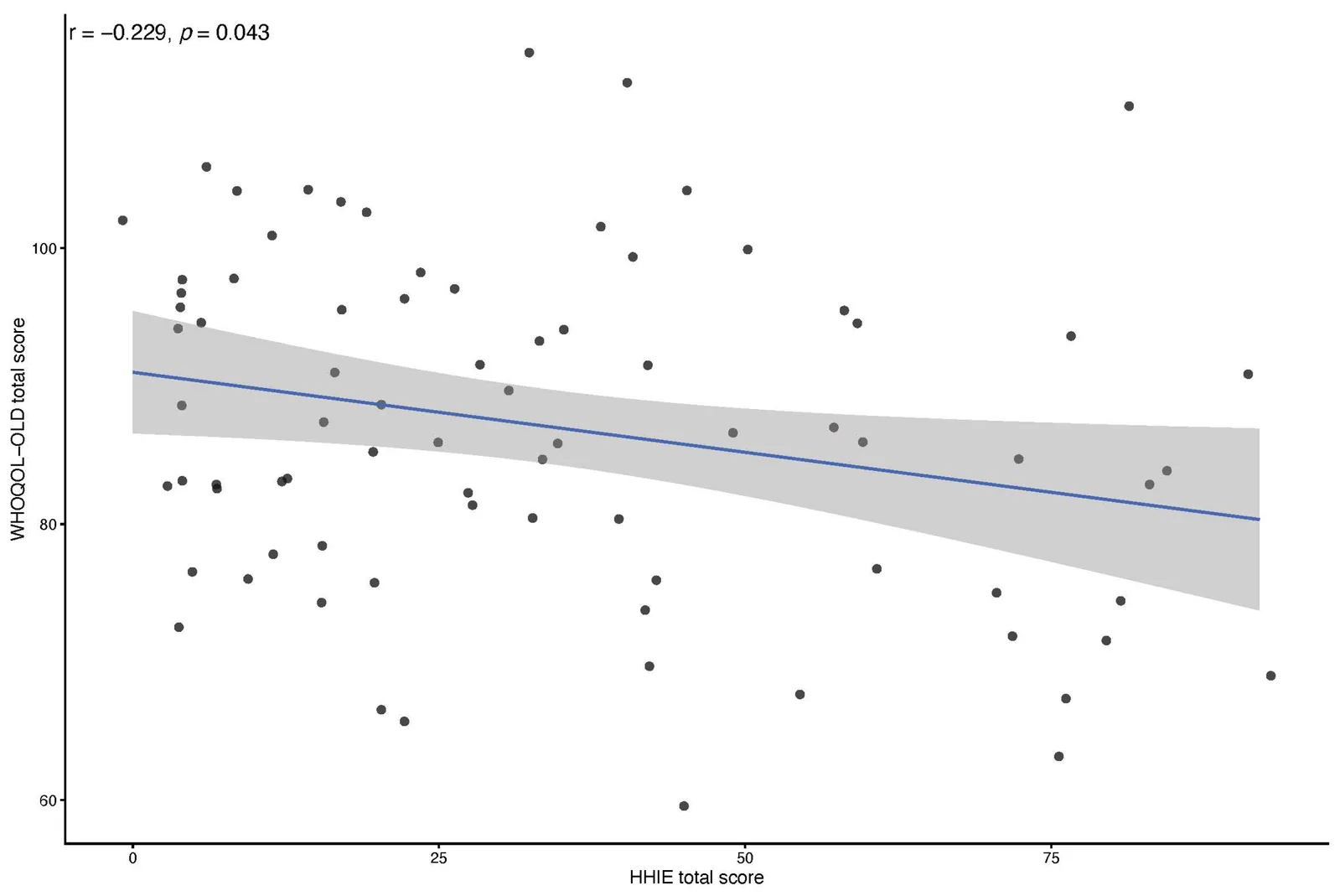
Scatterplot showing the association between HHIE total score and WHOQOL-OLD total score. Spearman’s correlation coefficient (r) and corresponding *p*-value are shown. The line represents the fitted trend with a 95% confidence interval. The grey shaded area represents the 95% confidence interval around the fitted regression line.

**Table 1:** Demographic, clinical, and audiological characteristics of the participants.

Characteristic	Category/Measure	Value
**Demographic characteristics**		
Sex	Female	32 (40.5%)
	Male	47 (59.5%)
Education level	No formal education	8 (10.1%)
	Primary school	43 (54.4%)
	Middle school	2 (2.5%)
	High school	15 (19.0%)
	University	11 (13.9%)
Marital status	Married	70 (88.6%)
	Divorced	2 (2.5%)
	Widowed	7 (8.9%)
Employment status	Employed	9 (11.4%)
	Retired or unemployed	70 (88.6%)
**Clinical characteristics**		
Tinnitus	Present	55 (69.6%)
	Absent	24 (30.4%)
Age, years	Median (IQR), min–max	66 (62–70), 60–78
Duration of hearing loss, years	Median (IQR), min–max	3 (1–7), 1–30
**Audiological measures**		
Right ear PTA, dB HL	Median (IQR), min–max	35.0 (25.0–47.5), 16.25–76.25
Left ear PTA, dB HL	Median (IQR), min–max	30.0 (21.88–44.38), 16.25–68.75
Better ear PTA, dB HL	Median (IQR), min–max	27.5 (18.75–38.12), 16.25–68.75
Better ear hearing loss severity	16–25 dB HL	34 (43.0%)
	26–40 dB HL	28 (35.4%)
	41–55 dB HL	16 (20.3%)
	56–70 dB HL	1 (1.3%)
Worst ear PTA, dB HL	Median (IQR), min–max	37.5 (27.50–50.00), 16.25–76.25
Worst ear hearing loss severity	16–25 dB HL	14 (17.7%)
	26–40 dB HL	29 (36.7%)
	41–55 dB HL	25 (31.6%)
	56–70 dB HL	10 (12.7%)
	>71 dB HL	1 (1.3%)
Symmetric hearing loss	n (%)	63/79 (79.7%)
Asymmetric hearing loss	n (%)	16/79 (20.3%)
Word recognition score in quiet, %	Median (IQR), min–max	88 (80–96), 56–100
Word recognition score at +10 dB SNR, %	Median (IQR), min–max	72 (54–84), 24–96
Word recognition score at 0 dB SNR, %	Median (IQR), min–max	32 (16–50), 0–76
**Hearing handicap measures**		
HHIE total score	Median (IQR), min–max	28 (12–50), 0–92
HHIE emotional	Median (IQR), min–max	14 (5–26), 0–52
HHIE situational	Median (IQR), min–max	16 (6–24), 0–44
**Quality of life measures**		
WHOQOL-OLD total score	Median (IQR), min–max	86 (77.5–96), 60–114
WHOQOL-OLD autonomy	Median (IQR), min–max	15 (13–17), 7–20
WHOQOL-OLD past, present, and future activities	Median (IQR), min–max	15 (13–17), 7–20
WHOQOL-OLD social participation	Median (IQR), min–max	15 (12.5–16), 5–20
WHOQOL-OLD intimacy	Median (IQR), min–max	16 (13.5–17.5), 9–20
WHOQOL-OLD death and dying	Median (IQR), min–max	15 (11–17), 4–20
WHOQOL-OLD sensory abilities	Median (IQR), min–max	14 (12–16), 6–20

Variables are presented as median (interquartile range) and minimum–maximum values. Categorical variables are presented as n (%). Abbreviations: PTA, pure-tone average; SNR, signal-to-noise ratio; HHIE, Hearing Handicap Inventory for the Elderly; WHOQOL-OLD, World Health Organization Quality of Life-OLD module.

**Table 2:** Hierarchical multiple linear regression models predicting HHIE total score.

WRS Condition Entered in Step 2	Predictor	B	SE	t	*p* Value	VIF	Final R^2^	Final Adjusted R^2^	ΔR^2^	F Change	*p* for ΔF
WRS in quiet	**Model fit**	—	—	—	—	—	0.259	0.208	0.001	0.121	0.729
	Age	0.173	0.543	0.319	0.750	1.052					
	PTA	0.608	0.296	2.056	0.043	2.044					
	Duration of hearing loss	1.234	0.500	2.470	0.016	1.196					
	Tinnitus, absent	−2.622	5.786	−0.453	0.652	1.043					
	*WRS in quiet*	−0.092	0.264	−0.348	0.729	1.966					
WRS at +10 dB SNR	**Model fit**	—	—	—	—	—	0.295	0.247	0.037	3.836	0.054
	Age	0.060	0.530	0.113	0.911	1.054					
	PTA	0.305	0.293	1.041	0.302	2.104					
	Duration of hearing loss	1.257	0.487	2.581	0.012	1.194					
	Tinnitus, absent	−2.778	5.578	−0.498	0.620	1.019					
	*WRS at +10 dB SNR*	−0.340	0.173	−1.958	0.054	1.930					
WRS at 0 dB SNR	**Model fit**	—	—	—	—	—	0.300	0.252	0.042	4.399	**0.039**
	Age	0.007	0.531	0.013	0.989	1.066					
	PTA	0.279	0.292	0.955	0.343	2.109					
	Duration of hearing loss	1.111	0.489	2.270	0.026	1.213					
	Tinnitus, absent	0.513	5.713	0.090	0.929	1.077					
	*WRS at 0 dB SNR*	−0.390	0.186	−2.097	0.039	2.085					

The base model included age, pure-tone average, duration of hearing loss, and tinnitus status. Each WRS variable was entered separately in Step 2. Coefficients and VIF values represent the final model after adding the relevant WRS variable. The base model explained 25.8% of the variance in HHIE total score, R^2^ = 0.258, adjusted R^2^ = 0.218. Tinnitus present was used as the reference category. All VIF values were below 2.2, indicating no evidence of problematic multicollinearity. As a sensitivity analysis for multiple testing across the three correlated WRS models, permutation-based max-F adjusted *p*-values were 0.964, 0.116, and 0.045 for WRS in quiet, +10 dB SNR, and 0 dB SNR, respectively. Bold values indicate statistical significance (*p* < 0.05). Abbreviations: HHIE, Hearing Handicap Inventory for the Elderly; WRS, word recognition score; SNR, signal-to-noise ratio; PTA, pure-tone average; SE, standard error; VIF, variance inflation factor.

## Data Availability

The data presented in this study are available on reasonable request from the corresponding author. The data are not publicly available due to privacy, legal, and ethical restrictions.

## Supplementary Materials

The following supporting information can be downloaded at https://www.mdpi.com/article/10.3390/audiolres16050143/s1. Table S1: Comparison of word recognition scores across listening conditions; Table S2: Spearman correlations of WRS with HHIE and WHOQOL-OLD, full sample versus non-ceiling subsample; Table S3: Hierarchical regression, incremental contribution of each WRS condition to HHIE total, full sample versus non-ceiling subsample.

## Abbreviations

The following abbreviations are used in this manuscript:

PTA	Pure-tone average
WRS	Word recognition score
SNR	Signal-to-noise ratio
HHIE	Hearing Handicap Inventory for the Elderly
WHOQOL-OLD	World Health Organization Quality of Life-Older Adults Module
SRT	Speech recognition threshold
MCL	Most comfortable level
MMSE	Mini-Mental State Examination
dB HL	Decibel hearing level
