# Supplementary material for: Associations of Word Recognition in Noise with Perceived Hearing Handicap and Quality of Life in Older Adults with Sensorineural Hearing Loss
Source: Audiol Res. 2026 Sep 25;16(5):143. doi: 10.3390/audiolres16050143 (PMC13701767; doi:10.3390/audiolres16050143)
Supplement: Supplementary file 1 [file audiolres-16-00143-s001.zip › audiolres-4534396-supplementary.pdf]

Supplementary Materials

Supplementary Table S1. Comparison of word recognition scores across listening conditions

Listening condition	Median (IQR)	Min–Max	Friedman χ^2	df	p value	Kendall's W	Post-hoc comparison	Adjusted p value
Quiet	88 (80–96)	56–100	157.50	2	**<0.001**	0.997	Quiet vs +10 dB SNR	**<0.001**
+10 dB SNR	72 (54–84)	24–96					Quiet vs 0 dB SNR	**<0.001**
0 dB SNR	32 (16–50)	0–76					+10 dB SNR vs 0 dB SNR	**<0.001**

Values are presented as median (interquartile range) and minimum–maximum. Friedman test was used to compare word recognition scores across the three listening conditions. Post-hoc pairwise comparisons were performed using Wilcoxon signed-rank tests with Bonferroni correction.
Abbreviations: WRS, word recognition score; SNR, signal-to-noise ratio; IQR, interquartile range.

Word recognition in quiet showed a ceiling effect, with 31 of 79 participants (39.2%) scoring at or above 96% and 10 (12.7%) reaching 100%. Such high scores were uncommon at +10 dB SNR (2 of 79, 2.5%) and did not occur at 0 dB SNR (maximum 76%). To test whether this influenced the findings, the correlation and hierarchical regression analyses were repeated after excluding participants who scored at or above 95% in quiet, leaving a subsample of 48 participants. Tables S2 and S3 compare the full sample with this subsample.

Supplementary Material Table S2. Spearman correlations of WRS with HHIE and WHOQOL-OLD, full sample versus non-ceiling subsample

WRS condition	HHIE total	HHIE emotional	HHIE situational	WHOQOL total	WHOQOL sensory
Quiet, full (n = 79)	-0.283*	-0.236*	-0.310**	-0.075	0.144
Quiet, subsample (n = 48)	-0.478***	-0.430**	-0.502***	0.092	0.213
+10 dB SNR, full	-0.452***	-0.426***	-0.439***	0.042	0.283*
+10 dB SNR, subsample	-0.584***	-0.559***	-0.560***	0.194	0.407**
0 dB SNR, full	-0.491***	-0.470***	-0.473***	0.118	0.313**
0 dB SNR, subsample	-0.559***	-0.535***	-0.566***	0.204	0.427**

Note: p < 0.05, p < 0.01, p < 0.001. The subsample excludes participants scoring at or above 95% in quiet.

Supplementary Material Table S3. Hierarchical regression, incremental contribution of each WRS condition to HHIE total, full sample versus non-ceiling subsample

WRS condition entered in Step 2	Sample	ΔR^2	F change	p	B (SE)
Quiet	Full (n = 79)	0.001	0.121	0.729	-0.092 (0.264)
Quiet	Subsample (n = 48)	0.020	1.286	0.263	-0.409 (0.361)
+10 dB SNR	Full (n = 79)	0.037	3.836	0.054	-0.340 (0.173)
+10 dB SNR	Subsample (n = 48)	0.120	8.898	0.005	-0.677 (0.227)
0 dB SNR	Full (n = 79)	0.042	4.399	0.039	-0.390 (0.186)
0 dB SNR	Subsample (n = 48)	0.121	9.006	0.005	-0.872 (0.290)

Note: Each WRS variable was entered separately in Step 2, after age, pure-tone average, duration of hearing loss, and tinnitus status. The subsample excludes participants scoring at or above 96% in quiet.
